# Bridging the Gap: Challenges and Innovations in Validating Next-Generation Sequencing Discoveries of Arboviruses

**DOI:** 10.3390/v18101094

**Published:** 2026-10-03

**Authors:** Kaiyue Wei, Xiaofeng Sun, Anum Farid, Hong Liu

**Affiliations:** 1 Center for Organoid and Genome Research, School of Life Sciences and Medicine, Shandong University of Technology, Zibo 255049, China; wky24410011008@outlook.com (K.W.); sunxiaofeng12345@outlook.com (X.S.); 2 School of Agriculture Engineering and Food Science, Shandong Dairy Technology Innovation Center, Shandong University of Technology, Zibo 255049, China; anum.malik911@gmail.com

**Keywords:** arbovirus, next-generation sequencing (NGS), virus isolation, biological validation

## Abstract

Next-generation sequencing (NGS) has dramatically accelerated the identification of viral genomic sequences from arthropod vectors, uncovering an unprecedented diversity of potential arboviruses. Nevertheless, the majority of these novel viral genomic sequences (NVGS) remain uncharacterised genomic data without corresponding viral isolates, highlighting the discrepancy between sequence detection and biological validation. This gap impedes our understanding of the biological properties, host range, and pathogenic potential of these newly identified viruses. To bridge this gap, the review advocates for integrated strategies, including expanded cell culture screening, novel cell line development, and reverse genetics approaches. These are complemented by artificial intelligence (AI) for predictive viral characterization. In parallel, NGS can be combined with other sequencing strategies to obtain more complete and accurate viral whole-genome sequences. Validating NGS discoveries is essential for effective risk assessment and outbreak preparedness against emerging arboviral threats, with important implications for public health surveillance.

## 1. Arbovirus and Arbovirus-Associated Diseases

Arboviruses (Arthropod-borne viruses) constitute a group of viruses transmitted by hematophagous arthropods, which can result in significant morbidity and mortality among humans and animals globally. By 1985, a total of 504 arboviruses had been isolated from blood-feeding insects, including mosquitoes, ticks, midges, and sandflies, with over 100 identified as causative agents of diseases in humans and animals [1,2]. Notably, all these 504 viruses were isolated using traditional cell culture techniques and demonstrated the ability to replicate in various insect or mammalian cell lines (such as C6/36, BHK, and Vero) or in animal models (such as suckling mice and chicken embryos), resulting in cytopathic effects (CPE) and animal mortality. The biological characteristics of these viral isolates, including viral titers, plaque morphology, replication dynamics, and structural features, have been extensively studied. Additionally, research has been conducted to assess the pathogenesis of each virus using relevant animal models and by measuring neutralizing antibodies against the virus in human serum [3].

**Figure 1:**
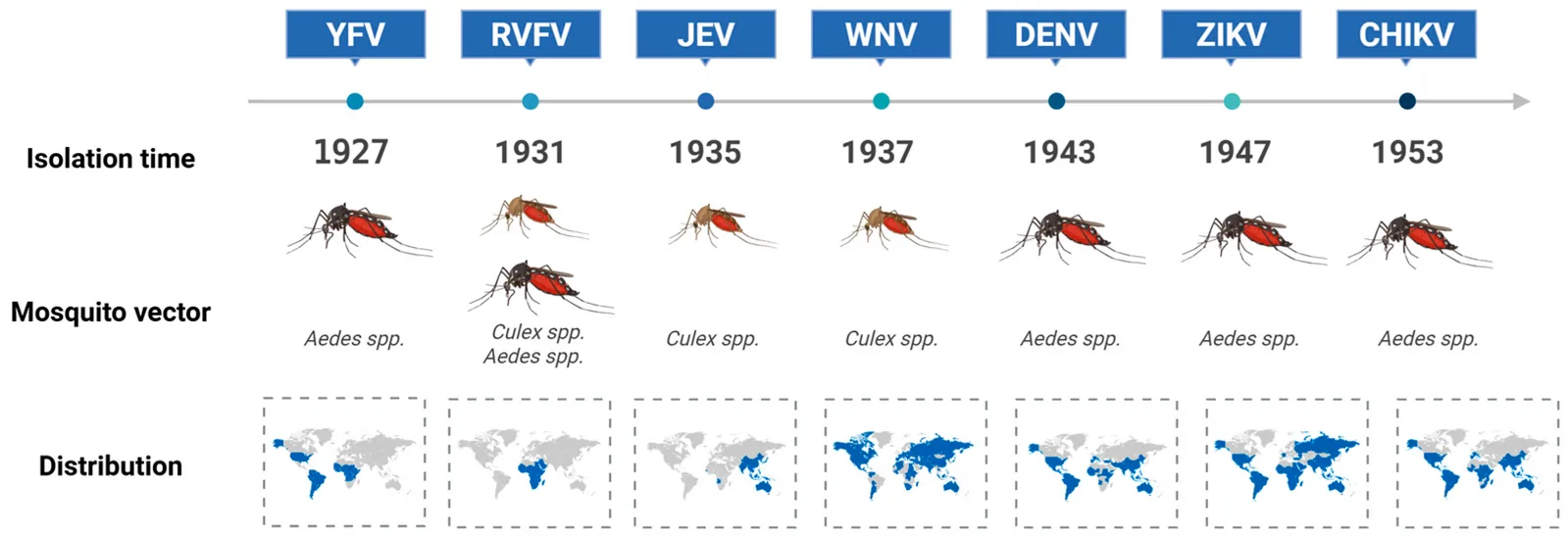
Global distribution, primary mosquito vectors, and initial isolation time of seven medically important arboviruses. YFV was first isolated in 1927 and is transmitted by *Aedes* spp. It is endemic/epidemic mainly in tropical regions of South America, Central America, and Africa. RVFV was first isolated in 1931 and is transmitted by *Culex* spp. and *Aedes* spp. It occurs mainly in Africa and the Arabian Peninsula. JEV was first isolated in 1935 and is transmitted mainly by *Culex* spp. It is endemic in temperate and tropical regions of South, Southeast, and East Asia and is widely distributed in the Western Pacific Region. WNV was first isolated in December 1937 from the blood of a woman in the West Nile District of Uganda and is transmitted by *Culex* spp. Its distribution now extends across much of Africa, parts of Europe, the Americas, the Middle East, West Asia, and Australia (Kunjin subtype). DENV was first isolated in 1943 and is transmitted by *Aedes* spp. It is endemic in more than 100 countries and territories, including Southeast Asia, the Pacific islands, Central and South America, and Africa. ZIKV was first isolated in April 1947 from the serum of a rhesus monkey in the Zika Forest of Uganda and is transmitted by *Aedes* spp. It is widely distributed in the Americas, the Western Pacific, Africa, Southeast Asia, and the Eastern Mediterranean Region, with an autochthonous case reported in Europe (Var Department, France). CHIKV was first isolated in 1953 and is transmitted by *Aedes* spp. It circulates extensively in Africa, the Americas, Southeast Asia, and the Western Pacific Region, causes intermittent outbreaks in the Eastern Mediterranean Region, and has led to sporadic autochthonous transmission in Southern Europe (Italy and France).

Over the past two decades, significant arboviruses and their associated infectious diseases that have achieved global prevalence—such as the Zika virus (ZIKV) [4], Rift Valley fever virus (RVFV) [5], dengue virus (DENV) [6], chikungunya virus (CHIKV) [7], yellow fever virus (YFV) [8], West Nile virus (WNV) [9], and Japanese encephalitis virus (JEV) [10]—were all isolated prior to 1985, with systematic investigations into their biological characteristics already undertaken [11]. Although large-scale outbreaks of these diseases had not occurred at the time of their initial isolation, the insights gained from these systematic studies on viral biology have been instrumental in the subsequent management and control of arboviral diseases. For example, during the 2015 Zika outbreak in South America, researchers promptly performed comparative molecular evolution analysis between newly isolated strains and the 1947 reference isolate [12], concurrently advancing diagnostic development, vaccine research, and antiviral discovery. Without the “live virus” isolates of the ZIKV from decades ago and the accumulated biological data, it would have been challenging to respond quickly to outbreaks of emerging arthropod-borne viruses like Zika and their associated diseases. This underscores the importance of having culturable “live viruses” for effectively addressing outbreaks of newly emerging arthropod-borne viruses (Figure 1).

## 2. Using Next-Generation Sequencing (NGS), a Large Number of Novel Viral Genomic Sequences Have Been Discovered

**Figure 2:**
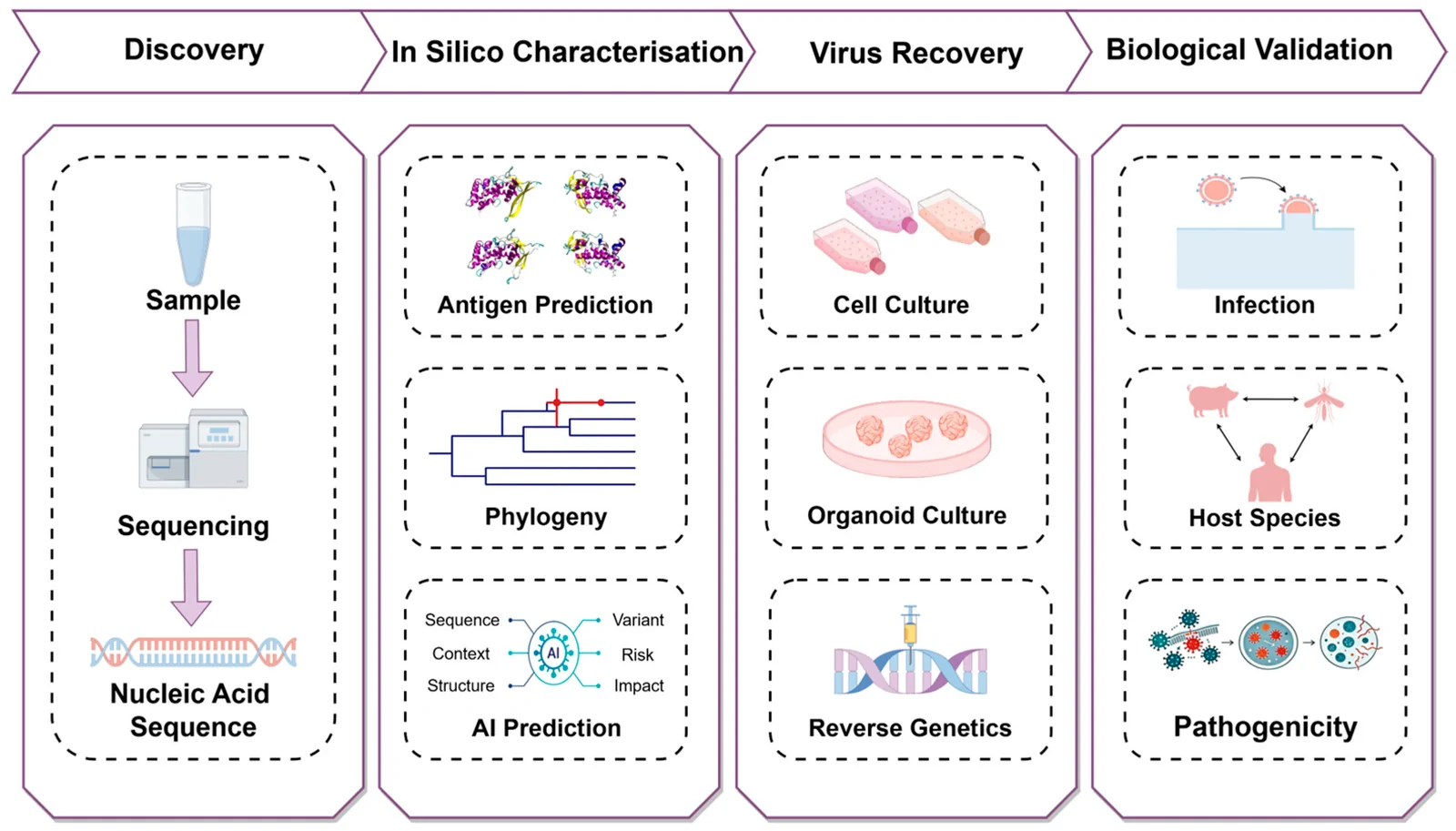
Schematic workflow of NVGS discovery and characterisation. The pipeline comprises four sequential stages: (1) Discovery—identification of novel viral sequences from NGS/metagenomic data; (2) In Silico Characterisation—bioinformatics analysis of antigen prediction, phylogeny and AI prediction; (3) Virus recovery—isolation or reconstruction of the virus through cell culture, organoid culture, and reverse genetics; (4) Biological validation—experimental confirmation of infectivity, host range, and pathogenicity of the newly identified sequences or isolates.

In recent years, many previously unknown novel viral genomic sequences (NVGS) have been discovered using next-generation sequencing (NGS) technology and bioinformatics methods, such as viromics and metatranscriptomics. For example, a recent metagenomic survey in Brazil’s Juruá River region identified >500 viral sequences from 18 families and 18 putative novel species, with strains spanning nine known viruses [13]. The application of NGS to mosquito and midge specimens has led to the identification of numerous novel members of the *Flaviviridae* family, in addition to nucleotide sequences corresponding to 180 *Monjiviricetes* viruses and 130 *Bunyavirales* viruses. NGS of bat specimens identified nucleotide sequences of 120 potential novel viruses [14]. Similarly, NGS of rodent specimens revealed 162 novel mammalian viral nucleotide sequences, including 24 with potential risks to humans [15]. There is no doubt that the vast amount of NVGS discovered through NGS technology has greatly enhanced our understanding of the diversity of viral species populations and the evolutionary patterns of viruses [16,17]. The rapid increase in NVGS, despite the fact that these sequences have not been validated by virus isolates, has significantly contributed to the availability of viral genomic sequences in public databases (such as NCBI), leading to substantial revisions in virus classification by the International Committee on Taxonomy of Viruses in 2021. This revision categorized animal viruses into five kingdoms, five subkingdoms, eleven phyla, twenty-six classes, thirty-six orders, and ninety-nine families [18]. The overall workflow for the NGS-based discovery of NVGS, spanning sample collection and sequencing, bioinformatic analysis, virus identification, database deposition, and taxonomic classification, is illustrated in Figure 2.

## 3. The NVGS Are Not Novel Viral Isolates and Have Inherent Limitations

**Figure 3:**
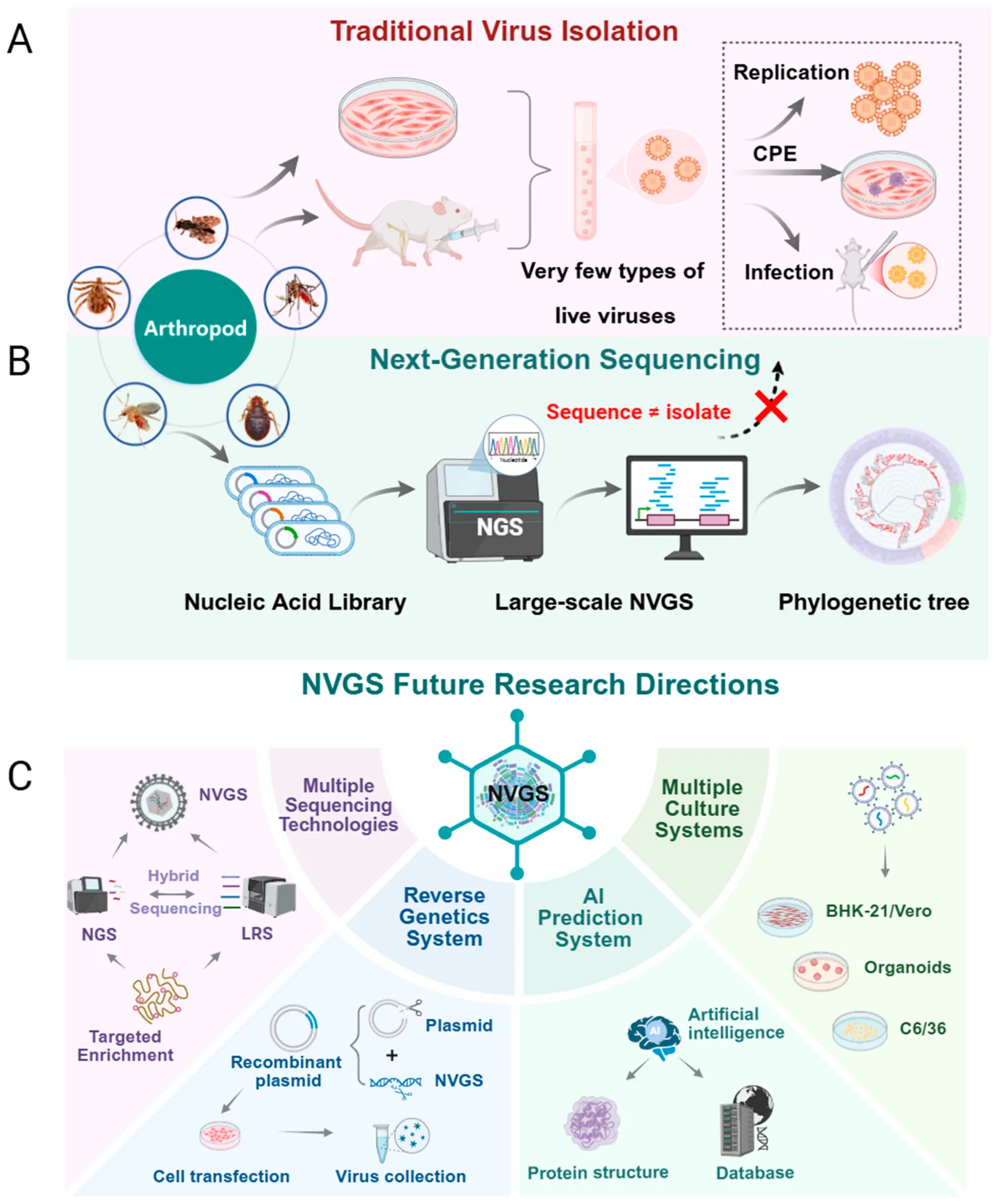
Evolution of Arbovirus detection. (**A**) Traditional cell culture techniques for viral isolation and identification. (**B**) The use of NGS has led to the discovery of a substantial number of novel viral genomic nucleotide sequences (NVGS). (**C**) Future research directions for NVGS.

In traditional virus isolation, samples collected from blood-feeding insects, such as mosquitoes, ticks, midges, and sand flies, are inoculated into susceptible cell lines, experimental mice, or embryonated chicken eggs for virus propagation [19]. Through this process, live virus strains with replicative capacity are obtained, and their replication efficiency, pathogenicity, and infectivity are systematically observed and assessed [20,21] (Figure 3A).

NGS is a high-throughput technology for rapidly determining nucleic acid sequences [22]. It involves fragmenting DNA (or RNA converted to cDNA) into short segments, commonly generating sequencing reads of approximately 150 nucleotides [23]. Subsequently, bioinformatics methods are used to assemble these nucleotide sequence fragments into contigs, which are classified into various “viruses” based on comparisons with already discovered viral genomic nucleotide sequences [24] (Figure 3B).

In fact, viral metagenomic NGS mainly provides sequence-based evidence for NVGS or candidate viral genomes, rather than experimentally validated viral isolates. Consequently, many viruses identified by this approach remain uncultured, leaving their key biological properties unknown, including infectivity, host range, replication characteristics, and pathogenicity [25]. These properties require further confirmation through virus isolation, targeted PCR validation, genome completion, or functional assays (Figure 3B). Moreover, the NVGS obtained via NGS are frequently partial genomic fragments rather than complete sequences that include non-coding regions, structural genes, and non-structural genes [26], limiting their capacity to systematically represent the full spectrum of viral genome features. The quality of NVGS is also highly dependent on sequencing platforms and downstream bioinformatics analyses. At the experimental level, common issues include host nucleic acid contamination, insufficient sequencing depth, and short read lengths [27,28]. At the analytical stage, when closely related reference sequences are unavailable, de novo assembly often produces chimeric contigs or misassemblies [28]. Furthermore, the absence of standardized quality control in virus-discovery workflows across laboratories undermines both the reliability and reproducibility of NVGS identification [29].

In summary, the NVGS identified by NGS are not derived from replicable “live viruses” or true viral isolates. They possess inherent limitations in sequence completeness, data quality, and biological representativeness. Consequently, they cannot directly provide critical biological information regarding viral infectivity, host range, replication dynamics, or pathogenicity [25]. This raises several fundamental questions about whether these sequences can assemble into functional virus particles, the biological phenotypes such particles would possess if they do, and their ability to infect host cells. More critically, it remains unknown whether these NVGS pose any risk of causing diseases in humans, animals, or plants, and what implications they may have for public health.

However, not all NVGS are of equal research value. When faced with a massive number of NVGS, researchers should establish a risk-oriented prioritization framework to rationally allocate validation resources [30,31]. We therefore propose an illustrative scoring logic to make this framework more operational. Each NVGS is scored against four criteria: A. phylogenetic relatedness to known human or livestock pathogens (0, distinct; 1, unknown; 2, closely related); B. exposure potential, i.e., detection in vectors that frequently bite humans (0, none; 1, uncertain evidence; 2, clearly detected); C. breadth of host range (0, single host; 1, unknown or two host orders; 2, three or more host orders); D. evidence of human or livestock infection (0, none; 1, indirect, uncertain, or from a single source; 2, direct and reproducible). Scores are summed, giving a total range of 0–8, which is then mapped to three priority tiers: a total of 6–8 indicates high priority, for which immediate biological validation is recommended; a total of 3–5 indicates medium priority, to be advanced stepwise after the genome has been fully assembled and further characterized; a total of 0–2 indicates low priority, to be retained for continuous surveillance and sequence accumulation. For example, a sequence closely related to a known pathogen (A = 2) but found in a wildlife host with no known human contact (B = 0, C = 1, D = 0) scores 3 and is tentatively classified as medium priority for continued surveillance, whereas a sequence phylogenetically distant from known pathogens (A = 0) but detected in vectors that frequently bite humans (B = 2), with host range and infection evidence still unclear (C = 1, D = 1), scores 4 and is likewise assigned medium priority. A score might rise further as evidence accumulates: if human infection is subsequently documented (D = 2), the total increases to 5 and the sequence remains medium priority; should the host range simultaneously expand to three or more host orders (C = 2), the total reaches 6, and the sequence is upgraded to high priority.

It should be emphasized that this scoring logic serves only as a conceptual aid and is not a validated quantitative tool. Several limitations should be noted. The four criteria are not fully independent: a score of D = 2 implies that some route of human exposure already exists, and such a score is therefore unlikely to co-occur with B = 0. In addition, the additive combination allows a single strong signal to be diluted by low scores on the other criteria. The weights assigned to each criterion will need to be adjusted as empirical data accumulate. Therefore, developing and validating an operational prioritization tool remains an important task for future work.

## 4. Strengthening the Research on the Biological Significance of NVGS Discovered Through NGS

Since the beginning of the 21st century, emerging and re-emerging infectious diseases caused by pathogens originating from wildlife and transmitted by blood-feeding insects have emerged globally every 2–3 years [32]. To prevent future pandemics, the World Health Organization (WHO) has proposed strengthening research on the top 10 zoonotic infectious diseases transmitted by animals and hematophagous arthropod vectors that pose the greatest threat to humanity, including Disease X [33]. In 2022, the WHO launched the Global Arbovirus Initiative, emphasizing the importance of monitoring, preventing, detecting, and responding to arboviruses transmitted by blood-feeding insects [34].

Furthermore, over the past 20 years, arboviruses such as ZIKV, DENV, and CHIKV transmitted by mosquitoes have spread widely across the globe, resulting in a significant disease burden [35,36,37,38]. Therefore, it is reasonable to anticipate that the next emerging infectious disease outbreak could still be caused by viruses transmitted by blood-feeding insects, particularly mosquito-borne arboviruses [39]. As a result, it is crucial to strengthen research on various viruses transmitted by blood-feeding insects and on the large number of “NVGS” discovered through NGS obtained from specimens of blood-feeding arthropods, as well as their biological phenotypes.

It is noteworthy that the NVGS identified by NGS harbor a wealth of genomic information on unknown pathogens, which may include the causative agents of Disease X. Previous studies have shown that a considerable proportion of NVGS are genetically closely associated with known highly pathogenic arboviruses, constituting a realistic candidate reservoir for Disease X. For example, the Yezo virus (YEZV) was first obtained from metatranscriptomic sequencing of ticks, and it encodes a vOTU domain exhibiting both deubiquitinase (DUB) and deISGylase activities, suggesting that it may possess innate immune suppression capabilities comparable to those of Crimean-Congo hemorrhagic fever virus (CCHFV) [40]. Similarly, the Tamdy virus (TAMV) was initially discovered in ticks through viromic sequencing in China and belongs to the same genus, *Orthonairovirus*, as the highly pathogenic CCHFV. Subsequently, Moming et al. successfully isolated TAMV from ticks in northwestern China and, through serological investigation, confirmed that the virus had infected the local population as early as 2007, with an IgG seropositivity rate of 24.14% among febrile patients [41]. These viruses are transmitted by ticks, which bite humans, livestock, and various wildlife species; they have been detected in human populations, domestic animals, and wildlife hosts, indicating a broad host range and an intact transmission chain, thereby providing ecological conditions for cross-species spillover. Moreover, they have been confirmed or are highly suspected to cause febrile illnesses in humans. However, their geographic distribution and pathogenic mechanisms remain poorly understood, and no specific vaccines or therapeutic interventions are currently available. Should they become widespread, they could impose a significant public health burden.

However, these viral genome sequences are not genuine virus isolates. Analysis of nucleotide sequences cannot substitute for the biological phenotypes represented by virus isolates, such as vector transmission characteristics, pathogenicity to host animals, and potential pathogenicity to humans [26,42]. To obtain this information, replicable virus isolates are essential. Therefore, it is critically important to strengthen research on the viruses represented by the NVGS identified through NGS from various blood-feeding arthropod specimens, as well as their biological phenotypes. Unfortunately, conventional cell culture methods often fail to yield virus isolates corresponding to these NVGS [26,42]. Therefore, it is necessary to employ multiple culture systems, AI prediction systems, reverse genetics systems, and multiple sequencing technologies to obtain virus isolates corresponding to NVGS (Figure 3C). The advantages, limitations, and feasibility of each strategy are summarized in Table 1.

### 4.1. Advancing Virus Isolation Strategies: Expanding from Conventional Cell Lines to Novel Culture Systems

For the isolation and culture of arboviruses associated with blood-feeding insect specimens, the two most commonly used cell lines are mammalian cells (Vero and BHK) and insect cells (C6/36) [66,67]. The former are widely used for the isolation and propagation of vertebrate-infecting arboviruses, whereas the latter are generally more permissive for mosquito-borne arboviruses and insect-specific viruses. Therefore, using both types of cell lines on the same batch of blood-feeding insect specimens can lead to the isolation of zoonotic viruses that are sensitive to mammalian cells, such as DENV, WNV, and JEV, which have significant public health implications. Additionally, it allows for the isolation of viruses sensitive to insect cells. A large number of vector-borne viruses have been isolated using these cell lines [11,68]. Indeed, the standard method for DENV isolation employs cell lines such as C6/36, Vero, or BHK-21 [69]; WNV isolation and passage also widely utilize Vero cells [70]; and routine isolation of JEV can similarly be performed using Vero, BHK-21, or C6/36 cell lines. Together, these applications demonstrate that such cell lines are fully operable as routine tools for vector-borne virus isolation [71].

Because cells vary in their susceptibility to viruses, using only one cell line for vector-borne virus isolation can easily lead to missing isolates that are sensitive to other cell lines [43,66]. Therefore, it is advisable to use both mammalian and insect cell lines in parallel for virus isolation from the same batch of specimens to increase the yield of viral isolates, which may include some viruses corresponding to NVGS [44]. This is particularly important for discovering new viruses represented by “NVGS” in often-overlooked blood-feeding insects (e.g., biting midges, sandflies, and blackflies). Furthermore, for specimens subjected to deep sequencing (including blood-feeding arthropods such as mosquitoes and biting midges, as well as bats and rodents), it is recommended to simultaneously inoculate them into tissue culture cells, so that both “NVGS” sequences and viral isolates can be obtained from the same batch. Clearly, using multiple cell lines is essential to maximize the number of viral isolates obtained [22,44,68].

To obtain virus isolates corresponding to NVGS, there is an urgent need to develop novel cell culture systems. These include emerging organoid models and cell lines derived from various human tissues, such as liver, lung, intestine, and neural tissues. Such systems would help isolate viruses with tissue tropism and human pathogenicity, particularly those corresponding to NVGS discovered by NGS in various blood-feeding arthropods, such as mosquitoes, midges, ticks, and sandflies [72,73,74]. NVGS may represent viruses with diverse biological phenotypes, and some may even belong to pathogens that are difficult to culture [26,42,75]. For example, there are “non-cytopathic viruses” (like HBV and HCV) or viruses that exist in both cytopathic and non-cytopathic forms (like bovine viral diarrhea virus) [76,77]. Therefore, the development of new culture cell lines requires collaboration among cell biologists, virologists, and other relevant professionals.

This approach has relatively low hardware requirements, as routine cell culture equipment and a BSL-2 laboratory are sufficient, making it the most feasible option in resource-limited settings and suitable as the primary choice for arbovirus isolation in most laboratories. However, it is important to recognize the inherent limitations of parallel cultures using multiple cell lines. When the viruses represented by NVGS fail to replicate in the aforementioned cell lines, or in cases of non-cytopathic viruses or low-titer samples, even expanding the panel of cell lines may not yield the target virus isolates, necessitating supplementary detection methods such as immunofluorescence or RT-PCR. In a surveillance of tick-borne viruses conducted in Japan, Inoue et al. detected an unknown flavivirus sequence by NGS, tentatively designated Sarayaka virus (SAYAV). However, the virus failed to propagate in any of the cell lines tested, and no isolate exhibiting cytopathic effect (CPE) was obtained [46]. This result indicates that traditional cell lines have significant limitations in isolating the viruses represented by NVGS, and that their culture outcomes may be systematically biased toward “culturable” virus groups while underestimating those that are difficult to culture.

### 4.2. Using AI to Generate Hypotheses and Inferences Regarding the Biological Characteristics of Viruses Represented by “NVGS”

AI-assisted analysis offers a useful strategy for inferring the potential biological characteristics of viruses represented only by NVGS, especially when infectious isolates are unavailable. South Bay virus (SBV), a tick-associated virus identified in *Ixodes scapularis* from the northeastern United States, has had its complete genome characterized [78]. Taxonomically, SBV belongs to the genus *Sabavirus* within the family *Nairoviridae*, whereas Crimean-Congo hemorrhagic fever virus (CCHFV) belongs to the genus *Orthonairovirus* in the same family [78,79]. Similar NVGS related to SBV, exhibiting over 80% genomic homology to SBV, have also been detected in ticks from Yunnan Province, China; however, attempts to isolate infectious virus in C6/36 and BHK cells were unsuccessful [80]. Therefore, the absence of viral isolates limits direct studies of infectivity, host range, and pathogenic potential. In this context, AI-based comparative genomics, protein-structure inference, and host-range inference can be used to generate testable hypotheses regarding the biological properties and public-health relevance of NVGS-represented viruses, but cannot replace experimental determination [81].

In several defined tasks, AI-assisted analysis has demonstrated useful inferential performance. A novel computational framework, VirHostPRED, based on protein language models (PLMs), employs an SVM-RBF classifier to predict the human infection risk of viral proteins on an independent test set, achieving an accuracy of 0.852 and an AUC of 0.914 [48,52]. AlphaFold, developed by DeepMind, can directly predict protein structures from amino acid sequences. By leveraging known structures, multiple sequence alignments, and transformer-based attention networks, it achieves near-experimental accuracy for many well-represented protein families [53]. The Big Fantastic Virus Database (BFVD) contains highly diverse structures of various viral kingdoms, covering more viral variance than existing resources, and is the largest repository of predicted viral structures to date [82]. Viro3D is a protein structure prediction database specifically built for human and animal viruses, covering over 4400 virus species, enabling efficient identification of distantly related homologous proteins and greatly expanding functional annotation and deep evolutionary studies of viral proteins [83]. Taken together, these tools indicate that AI-assisted analysis can already provide valuable preliminary inferences about viral properties at the sequence, structural, and functional levels.

Therefore, it is suggested to utilize AI technology to conduct inferential analyses of the viruses represented by NVGS, so as to generate hypotheses that can be experimentally tested. The first step would involve analyzing the genomic structure, encoded protein structures, and three-dimensional conformations of “model viruses” (e.g., the CCHFV mentioned above) within the same viral genus as those represented by NVGS, particularly focusing on key factors that could lead to infections in humans and animals, such as receptor-ligand interactions [52,84]. Subsequently, the sequences of NVGS viruses can be integrated into the research model to infer their possible biological characteristics, as well as their associations with different blood-feeding vectors and host animals in natural cycles. This approach would help identify the relationships between NVGS-represented viruses and their closest vectors and host animals, thereby inferring the potential impact of NVGS-represented viruses on public health.

However, it is necessary to clearly recognize the limitations of AI-assisted analytical techniques. Their outputs should be regarded as hypotheses to be tested rather than definitive conclusions. AI predictions are, in essence, dependent on statistical learning from existing sequence-phenotype associations, and their outputs represent reasonable extrapolations of known data patterns rather than direct revelations of unknown biological functions. Their reliability is directly constrained by the sequence space covered by the training data; for novel viruses lacking closely related references, the uncertainty of extrapolation results increases significantly [49,85]. Specific biological traits such as viral viability and infectivity are determined by the synergistic effects of multiple loci within the genome, and the impact of a single mutation on phenotype is highly dependent on its sequence context (i.e., epistasis). Such high-order combinatorial spaces far exceed the coverage of current experimental data, making reliable predictions difficult for existing AI models [50]. Meanwhile, structure prediction tools such as AlphaFold can only provide static three-dimensional conformations and are unable to capture dynamic processes such as receptor binding, membrane fusion, or pH-dependent conformational transitions, nor can they directly determine the actual infectivity of viral particles based on such static structures [53]. Therefore, what AI offers are reasonable inferences based on similarity; the value of AI simulation studies lies not in replacing experimental validation, but in prioritizing candidate directions for further investigation. Whether viruses represented by NVGS truly possess specific biological characteristics such as replication capacity and infectivity ultimately must be confirmed through virus isolation and culture, as well as in vitro and in vivo propagation assays [3].

Regarding the conditions for implementation, reliable AI inference depends on high-quality, broad-coverage, and well-annotated viral sequence databases, as well as adequately trained prediction models. When facing rare or novel viruses with limited sequence information, its reliability decreases markedly [51]. At the same time, the application of AI analysis requires practitioners with interdisciplinary expertise in bioinformatics and virology to carry out model selection, parameter tuning, and result interpretation. The widespread availability of cloud-based computing platforms and open-source models has made AI analysis far more accessible than experimental approaches, thus offering a practical and low-cost pathway for preliminary assessments of the biological properties of NVGS-represented viruses. However, the quality of predictions remains highly dependent on the quality of the upstream NVGS data; in settings where sequencing capacity is insufficient or public database sharing is limited, the practical utility of AI applications will be greatly diminished.

### 4.3. Obtaining Recombinant Viruses Represented by “NVGS” Using Reverse Genetics Techniques

As mentioned earlier, NGS-based virome studies have revealed a large number of novel viral genomic sequences (NVGS) from animal and arthropod specimens [86]. Many of these sequences are related to viral lineages of medical or veterinary importance, including bunyavirids, filovirids, flavivirus-related viruses, and Jingmenvirus-like viruses [87,88]. Therefore, NVGS detected within these high-priority viral lineages may provide important clues for identifying potentially emerging viruses. However, in many cases, the corresponding infectious isolates are unavailable, which restricts direct investigation of viral infectivity, host range, tissue tropism, and pathogenic potential [25]. This limitation is further complicated by the fact that many NVGS identified by NGS are incomplete and often represent only partial genomic regions, such as structural protein genes [25]. For biologically important NVGS with only partial sequence information, molecular virology-based surrogate systems, such as pseudoviruses, replicons, minigenomes, or chimeric models, may provide controlled experimental platforms for functional evaluation without relying on natural viral isolates [55,89]. For example, viral gene components (promoters, structural genes, non-structural genes, etc.) from “NVGS” can be recombined into “infectious clones” of closely related viruses (or viruses in the same genus), effectively “rescuing” the novel virus represented by “NVGS” (e.g., recombining the structural genes of SBV into the infectious clone backbone of CCHFV), followed by a series of studies on the biological phenotypes and host infection characteristics of the recombinant virus [56].

Its effectiveness in the arbovirus field has been fully validated. Full-length infectious cDNA clones of JEV and ZIKV have been successfully constructed using the bacterial artificial chromosome (BAC) system [54,90]. Infectious clones of dengue virus (DENV) have also been recovered using a yeast genetic system [60]. For highly pathogenic viruses such as CCHFV, researchers transfected plasmids carrying the viral S, M, and L segments into BSR-T7/5 and Huh7 cells. Under the action of bacteriophage T7 RNA polymerase, complementary RNA copies of each segment were generated, and through trans-complementation by the nucleoprotein and L polymerase, high-titer infectious virus was successfully recovered [91].

It should be noted that this strategy demands the highest level of professional expertise and institutional infrastructure among all the approaches discussed. Its implementation requires the availability of gene synthesis and high-throughput cloning platforms, infectious clone libraries, as well as cell and animal experimental systems, supported by a professional team with high-level molecular virology skills, and supplemented by institutional conditions including BSL-3 facilities, ethical approval, and biosecurity management. Nevertheless, because reverse genetics can produce “artificial recombinant viruses” that can be directly used for assessing infectivity, host range, and pathogenicity, the biological information it provides has irreplaceable value that other methods cannot offer. Therefore, it should be prioritized for NVGS with high public health risks, such as those closely related to known highly pathogenic agents or with pandemic potential, rather than being implemented indiscriminately for all NVGS [92].

However, this resource threshold raises an unavoidable question. Arboviral diseases are mainly prevalent in low- and middle-income countries, and NVGS are increasingly being discovered in these regions; yet the BSL-3 facilities, gene synthesis and high-throughput cloning platforms, and specialized personnel required for reverse genetics remain concentrated in high-income countries. As a result, high-risk viruses often circulate in settings with the weakest validation capacity, while institutions with the necessary technical capacity may not possess the most relevant local strains and epidemiological context. Addressing this situation requires international cooperation: establishing or strengthening BSL-3 facilities and cloning platforms in disease-endemic regions, promoting cross-institutional sharing of infectious clone libraries, cell resources, and computational capacity, and encouraging laboratories with the required capacity to conduct joint research with institutions in endemic regions. Cooperation should also be governed by pre-agreed collaborative frameworks to ensure that sequence data and the diagnostics, vaccines, and drug targets derived from them remain accessible to the source regions. In addition, rescue experiments involving highly pathogenic viruses must undergo ethical and biosafety review by the host country and collaborating parties.

The limitations and challenges of reverse genetics technology are equally prominent. Most NVGS are only partial genomic sequences, often lacking key elements such as terminal untranslated regions and replication/transcription signals required for constructing infectious clones, making it difficult to directly assemble full-length clones [59]. At the same time, sequence errors introduced during gene synthesis and cloning may introduce mutations inconsistent with the original NVGS, leading to rescue failure or phenotypic deviations. Moreover, chimeric viruses obtained by integrating heterologous structural genes into closely related viral backbones exhibit phenotypes resulting from complex interactions between the donor gene and the backbone viral genome. Therefore, inferences about the infectivity and pathogenicity of the original virus based on such chimeras must be made cautiously [57]. Most importantly, the generation of replication-competent recombinant viruses involves potential biosafety and dual-use risks, requiring operations to be conducted in biosafety level 3 (BSL-3) or higher facilities, and subject to ethical and biosecurity review.

### 4.4. Utilizing Multiple Sequencing Technologies to Refine the Genomic Structure of NVGS and Interrogate Quasispecies Diversity

Short-read sequencing on NGS platforms generates short fragments that make it difficult to resolve the complete gene structures, repetitive regions, and intra-host genetic heterogeneity of NVGS. Viruses often exist within a host or vector not as a single sequence but as a quasispecies—a population of closely related genetic variants differing by minor substitutions that arise during error-prone replication and provide the substrate for adaptive evolution [58,93]. Nevertheless, most current NVGS studies report only consensus sequences and rarely interrogate heterogeneity at the quasispecies level, thereby underestimating the biological information contained in the sequencing data [58].

The depth of analysis should therefore be matched to the research question and to the risk posed by the virus. For rapid screening and preliminary classification, consensus sequences are sufficient. For viruses closely related to known pathogens, or with pandemic potential, in-depth analysis of quasispecies diversity is warranted, because individual variants may differ in cell tropism, vector-transmission efficiency, or pathogenic potential. However, the short read lengths of conventional NGS prevent the linkage between distant variant sites from being determined reliably [94], which can produce chimeric haplotypes or underestimate diversity and impede accurate haplotype reconstruction [95].

Long-read sequencing technologies, exemplified by Oxford Nanopore Technologies (ONT) and Pacific Biosciences (PacBio), can sequence nucleic acids without PCR amplification, and a single read can span complex structures such as inverted terminal repeats (ITRs) and poly(A) tails. This markedly improves the resolution of complete viral genomes and provides linkage information across distant variant sites, thereby facilitating quasispecies haplotype reconstruction. For example, Phadphon et al. used ONT long-read sequencing to resolve the ITRs and the repeated inverted-complementary structures in the hairpin-loop region of African swine fever virus (ASFV), achieving full-length genome assembly [96]; similarly, Chang et al. used PacBio reads for the de novo assembly and annotation of herpes simplex virus type 2 (HSV-2), completing the genome of strain G [61]. It should be noted, however, that the comparatively higher raw error rates of long-read platforms, particularly ONT, complicate the reliable calling of low-frequency variants, and error correction (e.g., using HiFi reads or consensus polishing) is generally required.

Combining long- and short-read data (hybrid sequencing) exploits the complementary strengths of both approaches: long reads to span complex regions and phase variants, and short reads to provide per-base accuracy. In an evaluation of hybrid sequencing for SARS-CoV-2 genomic surveillance, Coşgun et al. found that hybrid assemblies were more complete than those obtained by Illumina sequencing alone, and that the identified variants were detected consistently and with higher reliability across methods [62]. Additional strategies can further increase the recovery of virus-derived sequence from clinical samples. Targeted enrichment captures target viral nucleic acids with specific probes or primers while depleting background non-target material, substantially increasing the proportion of viral reads even at very low viral loads [63]. Synthetic long-read approaches partition long DNA molecules into micro-reaction units, tag each unit with a unique molecular identifier (UMI), and then perform standard short-read sequencing; because fragments originating from the same long molecule share a UMI, the short reads can be computationally reassembled into long-range information, providing linkage data on short-read platforms [64]. These strategies can reconstruct viral genomes and their variant structure as completely as possible at the sequence level, providing reliable support for downstream isolation and culture, reverse genetics, and functional experiments.

Sequencing strategy should also be tailored to sample biology. Quasispecies diversity can differ by host origin, with vector-derived samples generally reported to harbour broader intra-host diversity than mammalian samples [65]; this should inform the choice of sequencing depth. When the predominant genetic characteristics of a viral population are of interest, a high-depth consensus sequence is sufficient; when adaptive evolution or minority-variant reservoirs are the focus, systematic low-frequency variant detection is required. To ensure comparability across host origins and platforms, a unified variant-detection threshold and strict quality-control procedures should be applied, and sequencing depth should be sufficient to capture low-frequency variants reliably in high-diversity samples such as those from insect vectors. Bioinformatic subtraction of host sequences and targeted viral enrichment can further focus the analysis on virus-derived reads.

Despite these advances, several limitations remain: the raw error rates of long-read sequencing, the lack of standardized algorithms for hybrid assembly, the difficulty of distinguishing true low-frequency variants from sequencing noise, and the unclear relationship between quasispecies diversity and viral phenotype. Importantly, sequencing provides more complete genomic information, but it cannot by itself verify infectivity or pathogenicity. Whether a virus is replication-competent, can infect specific host cells, or is pathogenic can only be determined through virus isolation, rescue of infectious clones, and in vitro and in vivo replication experiments. Thus, the role of sequencing is to provide complete full-length genomes for other validation strategies, which is a prerequisite for constructing infectious clones and performing reverse genetics; quasispecies-level information, in turn, can be used to identify haplotypes with variants at key functional sites, thereby defining the specific targets for functional testing and avoiding the indiscriminate testing of all variants one by one. Future work should establish cross-platform quality-control standards and translate quasispecies information into early-warning indicators for risk assessment, thereby linking sequencing results with downstream functional validation.

## 5. Conclusions

NGS has transformed virus discovery, but the sequences it yields are not viral isolates [25]. Most represent partial genomic fragments, and the biological properties that determine risk cannot be inferred from sequence alone [92]. Bridging this gap requires a tiered and integrated strategy rather than a breakthrough in any single technology.

In the short term, strategies including long-read, hybrid sequencing, and targeted enrichment should be integrated to establish a standardized NVGS generation pipeline, systematically completing existing fragmented sequences and constructing a public database of full-length NVGS covering vector, host, and geographic diversity [97].

In the medium term, the key bottlenecks in validation should be addressed: establishing high-throughput virus isolation systems using organoids and novel cell lines; developing automated and standardized pipelines for infectious clone construction and reverse genetics; and establishing a closed loop between AI prediction and experimental validation, in which experimental data refine predictive models and model output guides experimental design. In parallel, cross-laboratory quality control and benchmarking networks should be established.

In the long term, validated NVGS should be incorporated into global vector-borne pathogen surveillance systems, so that risk signals at the sequence level can be rapidly translated into candidate targets for vaccines, diagnostic reagents, and control strategies. This will require international mechanisms for sharing infectious clone platforms, cell resources and computational capacity, with agreed terms of access and benefit-sharing, so that preparedness against future arboviral epidemics, including Disease X, rests on validated rather than inferred viral diversity.

## Figures and Tables

**Table 1:** Advantages, Limitations, and Feasibility Assessment of the NVGS Research Strategy.

Strategy	Advantages	Limitations	Feasibility
**Section 4.1 Advancing virus isolation strategies: expanding from conventional cell lines to novel culture systems.**	Increases the virus isolation rate [43]; Low hardware requirements.	Using only a single cell line readily loses isolates sensitive to other cell lines [44,45]; Non-cytopathic, low-titer, or difficult-to-cultivate viruses may remain non-isolable even when the cell panel is expanded [46,47]; Outcomes tend to be biased toward “culturable” viruses.	Most feasible (It can be completed in a BSL-2 laboratory).
**Section 4.2. Using AI to generate hypotheses and inferences regarding the biological characteristics of viruses represented by “NVGS”.**	Enables inference of virus host range, protein structure, infection risk, and other biological characteristics without a viral isolate [48]; Low cost and easy access via cloud computing and open-source models.	Outputs are hypotheses only, not definitive conclusions [49,50]; Epistatic effects are difficult to predict [51]; Still requires virus isolation and in vitro/in vivo validation; Prediction quality is constrained by databases.	Relatively high feasibility, good practical accessibility, and low cost; Prediction quality depends on well-annotated, broad-coverage viral sequence databases and high-quality upstream NVGS data; Empirically supported, e.g., VirHostPRED (accuracy 0.852, AUC 0.914) [52,53].
**Section 4.3 Obtaining NVGS-represented recombinant viruses using reverse genetics techniques.**	Yields infectious recombinant viruses that allow direct evaluation of infectivity, host range, and pathogenicity [54]; For partial sequences, surrogate systems (e.g., pseudovirus, replicon, minigenome, and chimeric models) can be used for research [55,56].	Most NVGS are only partial sequences, lacking the UTRs and replication or transcription signals required to construct full-length clones [57]; Gene-synthesis/cloning errors and chimeric-backbone interactions can cause rescue failure or phenotypic deviation [58]; There are biosafety and ethical risks.	Low feasibility: highest professional and facility requirements (BSL-3); Fully validated in the arbovirus field (JEV, ZIKV, DENV, CCHFV, etc.) [54,59,60].
**Section 4.4 Utilizing multiple sequencing technologies to refine NVGS genomic structure and mine quasispecies information.**	Long-read sequencing (ONT/PacBio) spans complex and terminal structures, directly reflecting quasispecies heterogeneity [61,62]; Hybrid sequencing, targeted enrichment, and synthetic long-read strategies improve genome completeness and reliability [63,64,65].	High inherent error rate of long-read sequencing; Low-frequency variants are hard to distinguish from sequencing noise; the link between quasispecies diversity and viral phenotype remains unclear; Cannot replace virus isolation.	Moderate feasibility; depends on specialized platforms and unified quality control.

## Data Availability

The original contributions presented in the study are included in the article; further inquiries can be directed to the corresponding authors.
